# Oxygen uptake kinetics and biological age in relation to pulling force and 400-m front crawl performance in young swimmers

**DOI:** 10.3389/fphys.2023.1229007

**Published:** 2023-10-06

**Authors:** Marek Strzała, Kamil Sokołowski, Łukasz Wądrzyk, Robert Staszkiewicz, Łukasz Kryst, Magdalena Żegleń, Piotr Krężałek, Marcin Maciejczyk

**Affiliations:** ^1^ Department of Water Sports, Faculty of Physical Education and Sport, University of Physical Education, Kraków, Poland; ^2^ Department of Biomechanics, Faculty of Physical Education and Sport, University of Physical Education, Kraków, Poland; ^3^ Department of Anthropology, Faculty of Physical Education and Sport, University of Physical Education, Kraków, Poland; ^4^ Pain Research Group, Institute of Psychology, Jagiellonian University in Krakow, Kraków, Poland; ^5^ Department of Physiotherapy, Faculty of Motor Rehabilitation, University of Physical Education, Kraków, Poland; ^6^ Department of Physiology and Biochemistry, Faculty of Physical Education and Sport, University of Physical Education, Kraków, Poland

**Keywords:** aerobic power, oxygen uptake, tethered swimming force, front crawl, maturity

## Abstract

**Background:** The study aimed to assess differences in the biological age (*BA*) of 13-year-old swimmers and show their ability, as biologically younger—*late mature* or older—*early mature*, to develop fast 60-s oxygen uptake ( ${\dot{V}O}_{2}$ ) kinetics and tethered swimming strength. Furthermore, the interplay between swimming strength, ${\dot{V}O}_{2}$ , and 400-m front crawl race performance was examined.

**Methods:** The study involved 36 competitive young male swimmers (metrical age: 12.9 ± 0.56 years). Depending on *BA* examination, the group was divided into *early-mature* (*BA*: 15.8 ± 1.18 years, *n* = 13) and *late-mature* (*BA*: 12.9 ± 0.60 years, *n* = 23) participants, especially for the purpose of comparing tethered swimming indices, i.e., average values of force (*F*_ave_) and ${\dot{V}O}_{2}$ (breath-by-breath analysis) kinetic indices, measured simultaneously in 1-min tethered front crawl swimming. From the 400-m racing stroke rate, stroke length kinematics was retrieved.

**Results:** In the 1-min tethered front crawl test, *early-mature* swimmers obtained higher results of absolute values of ${\dot{V}O}_{2}$ and *F*_ave_. Conversely, when ${\dot{V}O}_{2}$ was present relatively to body mass and pulling force (in ml∙min^–1^∙kg^–1^∙N^−1^), *late-mature* swimmers showed higher $O_{2}$ relative usage. *Late-mature* swimmers generally exhibited a slower increase in ${\dot{V}O}_{2}$ during the first 30 s of 60 s. ${\dot{V}O}_{2}$ , *F*_ave_, *BA*, and basic swimming kinematic stroke length were significantly interrelated and influenced 400-m swimming performance.

**Conclusion:** The 1-min tethered swimming test revealed significant differences in the homogeneous calendar age/heterogeneous *BA* group of swimmers. These were distinguished by the higher level of ${\dot{V}O}_{2}$ kinetics and pulling force in *early-mature* individuals and lower efficiency per unit of body mass per unit of force aerobic system in *late-mature* peers. The higher ${\dot{V}O}_{2}$ kinetics and tethered swimming force were further translated into 400-m front crawl speed and stroke length kinematics.

## Introduction

The performance of young athletes training in competitive swimming depends on their circulatory and respiratory abilities, skeletal muscle energetics, and somatic traits, which are developed simultaneously with maturation and training. Therefore, testing and then proper training with appropriate intensity, corresponding to physiological changes, supporting the current development of the body and specific motor skills, constitute a priority. In addition, acting in a harmonious manner ensures long-term progress focused on the development of an adult elite swimmer (Pichardo et al., 2018).

In young swimmers, in the period of building up the foundations of endurance, aerobic capacity assessed as maximal oxygen uptake ( ${\dot{V}O}_{2}\max$ ) is the key to successful conditioning (Olbrecht, 2015). In the swimmer’s energy profile, the ability of the body to produce energy from aerobic vs. anaerobic metabolism in 100-m races, considered sprints in swimming, can be more than half (Hellard et al., 2018). Maximal aerobic power assessed in different ${\dot{V}O}_{2}$ protocols is among the best predictors of swimming aptitude and concerns the most common Olympic 200-m races. Based on the latest reports, it can be concluded that the protocols for assessing aerobic capacity include a graded test procedure, with the measurement of the anaerobic threshold and then reaching ${\dot{V}O}_{2}\max$ (Sokołowski et al., 2021). Other tests, most often performed in 400-m all-out swimming, assessed fast ability and also the fast component of reaching ${\dot{V}O}_{2}\max$ during heavy or severe intensities (Costill et al., 1985; Costill et al., 1992; Lätt et al., 2009; Zacca et al., 2019). In swimming competitions, the aerobic energy system participates in the overall energy production from the first part of the all-out swimming effort of 400-m (Rodríguez et al., 2003), 200-m (Figueiredo et al., 2011; Sousa et al., 2011), and even 100-m races (Ribeiro et al., 2015; Hellard et al., 2018) or 60 s (Sokołowski et al., 2022a). To control ${\dot{V}O}_{2}$ toward its peak, close to its maximum at 60 s (Gastin and Lawson, 1994), such extreme efforts are used for swimmers’ conditioning evaluation.

In competitive swimming, developing the fast component of ${\dot{V}O}_{2}$ through intensive aerobic training is sustainably accompanied by the conditioning of muscle strength needed for a swimmer to generate propulsion in appropriate stroking, which is at last manifested by a correspondingly longer stroke length (*SL*) and stroke index (*SI*) (Hellard et al., 2018). The ability of 13-year-old swimmers to develop fast 60-s ${\dot{V}O}_{2}$ kinetics can be useful in races longer than 100 m, i.e. 200-m (Sokołowski et al., 2022a) or 400-m races, enabling them to open the race with more ease by reducing the initial oxygen deficit and simultaneously reducing excessive fatigue.

During exercise, the fast phase of ${\dot{V}O}_{2}$ response to transition from rest to exercise may be shorter in trained than in untrained persons; the shorter time constant (*τ*) for the fast phase of ${\dot{V}O}_{2}$ was linked with better exercise performance (Arend et al., 2021). A significant positive correlation between the fast component and ${\dot{V}O}_{2}\max$ was observed (Ingham et al., 2007; Arend et al., 2021), but previous studies also reported no relationship between the fast component and ${\dot{V}O}_{2}\max$ (Pringle et al., 2003; Reis et al., 2012). The differences in fast ${\dot{V}O}_{2}$ , as evidenced in peer swimmers competing together in the same race, can be seen as one of the deterministic factors useful for practitioners. These differences may be noted not only in elite adult swimmers; it is equally interesting to see how they are exhibited within a given age group, as growth, development, and maturation may be masked or may be more intensely expressed than those caused by training (Baxter-Jones et al., 1993). Perhaps it is very difficult to completely separate the effects of maturation and training on the development of swimming skills. Nevertheless, one can assess the relationship between the biological age (*BA*) and the level of features determining swimming ability, which can be developed with appropriate intensity at the age of puberty and later (Strzała et al., 2019; Zacca et al., 2019; Fiori et al., 2022).

Nevertheless, to gather useful results to evaluate young swimmers’ strength potential and physiological endurance, tethered swimming can be used as a valid and reliable tool to measure exerted forces in water, where simultaneously physiological variables are not significantly different from free swimming effort of similar duration (Morouço et al., 2014; Ruiz-Navarro et al., 2020).

This study aimed to assess differences in *BA* of 13-year-old swimmers and show their ability, as biologically younger or older, to develop fast 60-s ${\dot{V}O}_{2}$ kinetics and tethered swimming strength. In addition, the interplay between swimming strength and ${\dot{V}O}_{2}$ was examined. Furthermore, the impact of these indices on performance in a 400-m front crawl race was determined, including stroke kinematics. It can be hypothesized that *BA* is a factor making a difference in fast ${\dot{V}O}_{2}$ kinetics and strength development, with further influence on swimming performance.

## Materials and methods

### Participants

Overall, 36 young male swimmers volunteered to participate in the study. Their metrical age was 12.9 ± 0.56 years, and their *BA* was estimated as 14.0 ± 1.56 years, with a body mass index of 19.1 ± 1.94 kg m^–2^. All swimmers were licensed by the Polish Swimming Federation, and their training experience was 4–5 years. Their swimming level according to FINA scores in the 400-m freestyle race was 323.0 ± 66.88 pts, which situates them at the fifth threshold in the Ruiz-Navarro et al. (2022) classification of the competitive level. The subjects performed 10 training sessions each week, versatile in style, with a load adapted to their age, and regularly competed in swimming competitions within their age group, including at the national level (Polish championships).

### Oxygen uptake and pulling force measurements in the tethered swimming test

**FIGURE 1:**
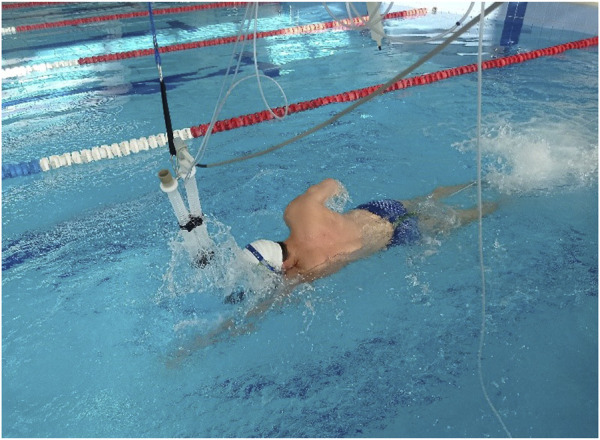
One of the participants during simultaneous oxygen uptake and tethered force measurements in the 1-min front crawl tethered swimming test.

The maximum front crawl tethered test was performed for 1 min, along with the measurement of ${\dot{V}O}_{2}$ and pulling. The test was carried out in an indoor swimming pool used for training and swimming competitions. A tether with a force gauge (ZPS5-BTU1kN, Staniak, Poland) was attached to the starting platform (the fixing point was 0.47 m above the water surface and throughout 3.7 m steel to the participants tied by a nylon belt). A force gauge recorded the pulling force at 100 Hz and transferred the data to a computer program for further analysis (MAX6v0M software, Poland). During this test, the swimmers breathed through a system of breathing valves in combination with an ergospirometer (Start 2000 MES, Poland). The expired air testing system was suspended under a specially designed mobile crane (Figure 1). Breath-by-breath analysis was conducted using software (Ergo 2000M software MES, Poland), and data were saved for further analysis. This method of measuring ${\dot{V}O}_{2}\max$ has been proved to be reliable (Neiva et al., 2017). A few days before the test, the swimmers were informed about the test procedure and requested to rest and maintain a normal diet the day before the test. After a 1000-m warm-up as usually applied before competition and having familiarized themselves with the instrumentation, the subjects performed the test in accordance with the procedure previously described by Sokołowski et al. (2022a) and Morouço et al. (2014). The all-out tethered test starts at a low intensity to avoid the inertial effect of the first strokes. The test began and ended with a loud whistle. The saved breath-by-breath data provided the basis to calculate the average ${\dot{V}O}_{2}$ in each 10-s period of the 1-min tethered test, e.g., ${\dot{V}O}_{2}$ _1-10_ for the first 10 s and ${\dot{V}O}_{2}$ _51-60_ for the sixth 10 s. Furthermore, indices of the entire 1-min test oxygen consumption ( ${\dot{V}O}_{2}$ _1-60_) and for the last 30 and 20 s ( ${\dot{V}O}_{2}$ _31-60_ and ${\dot{V}O}_{2}$ _41-60_, respectively) were calculated. The average value of force in the entire test (*F*_ave1-60_ [N]) was also determined for 10-s periods, e.g., *F*_ave1-10_ for the first 10 s and *F*_ave 51-60_ for the sixth 10 s. The swimmers’ relative ${\dot{V}O}_{2}$ in 10-s periods was calculated using their body mass and *F*_ave_ (ml∙min^–1^∙kg^–1^∙N^−1^). The average stroke rate (*SR*) was calculated based on the stroke cycles recorded during the 60-s period using a graph created by the software for each swimmer.

### 400-m front crawl race

The 400-m freestyle competition with the participation of judges was carried out in a 25-m swimming pool (all swimmers used the front crawl technique). An automatic timing device (Omega, Switzerland; OCP5, StartTime V) was applied. Before the race, the participants completed a 1000-m warm-up as in a competition. All trials were recorded using a camera at 50 Hz framing (GC-PX100BE, JVC, Japan). The camera was placed on a tripod at the bleachers, 7 m above the water surface, on an extension in the middle point of the pool.

The 400-m race surface swimming speed (i.e., clean surface front crawl swimming, *V*_surf_ [m·s^–1^]) was distinguished for a 13-m sector for each 25-m lap, besides the 10-m sector on the first lap. Times for separate sectors were measured when the swimmer’s head crossed the imaginary line linking the markers on both sides of the pool. Markers were placed at the side of the pool to indicate the lines separating three zones: (a) I 7-m turn zone (including the first 10-m start zone), (b) surface swimming zone (13 m), and (c) II turn zone (5 m). Times were measured when swimmers’ heads crossed the imaginary line linking markers at the sides of the pool (Kinovea ver. 0.8.15 software). The 400-m front crawl velocity (*V*_total_ [m·s^–1^]) was defined as 400 divided by the final time of the race. The video analysis and computation of the basic kinetic and kinematic indices were performed as previously described by Sokołowski et al. (2021).

### Kinematic indices

For the kinematic analysis, *SR*, *SL*, and *SI* were calculated. *SR* was defined as the number of full stroke cycles performed within a unit of time (in cycles∙min^–1^) and was calculated with the video analysis of three consecutive stroke cycles (intraclass correlation of 0.99, 95% CI: 0.960–0.997). *SL* was defined as the horizontal distance that the body traveled during a full stroke cycle and was calculated as

$$SL=\frac{v}{SR},$$ (1)

where *SL* (in m) is the stroke length, *v* is the swimming velocity, and *SR* is the stroke rate. Finally, *SI* was deemed as an overall swimming efficiency estimator and computed as

SI=SL∙v, (2)

where *SI* (in m^2^∙s^–1^) is the stroke index, *SL* is the stroke length, and *v* is the swimming velocity.

### Biological age

**FIGURE 2:**
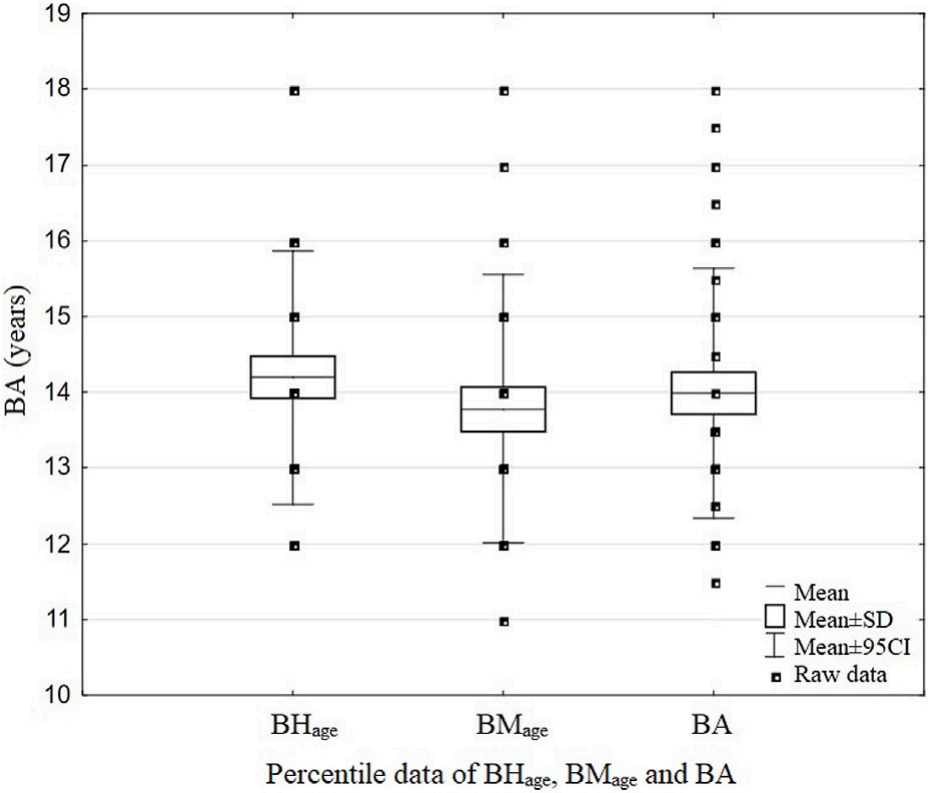
Data of *BHage*, *BMage*, and *BA* of the examined swimmers.

The swimmers’ *BA* was assessed based on the collected measurements of height (using a stadiometer, Sieber Hegner Maschinen AG, Switzerland) and body mass (Tanita BC-418, Japan). Then, age calculations were performed on the basis of the percentile charts for these data (Figure 2). The following formula was used:

$$BA=\frac{\left(BHage+BMage\right)}{2},$$ (3)

where *BHage* is the age obtained from the percentile charts in accordance with the participant’s body height and *BMage* is the age obtained from the percentile charts in accordance with the participant’s body mass. Growth charts by the Children’s Memorial Health Institute, standardized and validated for the Polish population, were used (the 50th percentile was applied to align height and mass with age). Additionally, pubertal development was assessed. Tanner stages based on pubic hair scale were estimated (Bornstein, 2018). Tanner’s scale was used only as an auxiliary measurement because the determination of biological sex relied mainly on the morphological criterion; Tanner’s scale was used in the event of three participants caused by large discrepancies in height- and weight-based ages.

*BHage*, age obtained from the percentile charts in accordance with the participant’s body height; *BMage*, age obtained from the percentile charts in accordance with the participant’s body mass; *BA*, biological age.

In this study, older—*early mature* (*BA*: 15.8 ± 1.18 years, calendar age: 13.0 ± 0.49 years) and younger (*BA*: 12.9 ± 0.60 years, calendar age: 12.8 ± 0.58 years) *BA* groups were also distinguished to show the differentiation of variables ( ${\dot{V}O}_{2}$ and *F*_ave_) obtained in the 10-s periods of the 1-min tethered test. The *early-mature* group involved 13 swimmers (biologically aged 14–18 years); the younger *BA* group—*late mature*—included 23 swimmers (biologically aged 11.5–13.5 years). Such an unequal division was due to the largest number (nine swimmers) of athletes aged 13.5, who were all placed in the late maturing group along with the even biologically younger swimmers.

### Statistical analysis

The assumption of the normality of the variables was verified by a visual examination of the distributions and the Lilliefors test. The results of this analysis were empowering to carry out a two-factor ANOVA (6 × 2) with 1 within-subject factor (six measurements of ${\dot{V}O}_{2}$ or *F*_ave_) and 1 between-subject factor (two *BA* groups: *early mature*, *n* = 13 and *late mature*, *n* = 23). The main effects of the measurements and the groups, as well as the interaction between them and simple effects, were calculated. In these calculations, the Greenhouse–Geisser correction was applied owing to the failure to meet the sphericity assumption. In the case of a simple comparison between the groups of biologically *early-mature* and *late-mature* swimmers for calendar age and *BA*, the independent sample Student’s *t*-test was applied. Pearson’s correlation coefficient was also computed between all variables collected during the 1-min tethered test and swimming kinematics or swimming speed for all swimmers (*n* = 36).

The analyses were performed for small, medium, and large effect sizes. An alpha level of 0.05 was assumed in all analyses. As for ANOVA, the power to detect significant interaction between a grouping factor and the within-subjects factor, three effect sizes were assumed: small: f = 0.10, medium: f = 0.25, and large: f = 0.40 (Cohen, 1988). The achieved power for the sample of 36 participants was 0.30, 0.98, and 0.99, respectively. For correlations, the assumed effect sizes were r = 0.10, 0.30, and 0.50. The achieved sample power value was 0.09, 0.45, and 0.92. In summary, for ANOVA, the sample size was satisfactory for medium and large effects but not for small effects. For correlations, the sample size was only enough to detect large effects. All statistical analyses were conducted using Statistica 13.3 software (TIBCO Software Inc., Palo Alto, CA, United States).

## Results

In this study, the division of swimmers into *early-mature* (older *BA*) and *late-mature* (younger *BA*) groups by presenting the most important variables ( ${\dot{V}O}_{2}$ and *F*_ave_), which explained their impact on swimming results, was justified. The groups did not differ significantly in terms of date of birth (t = −1.27, *p* = 0.213, Cohen d = 0.44); however, for these groups (*early mature*, *n* = 13 and *late mature*, *n* = 23), *BA* was significantly different (t = −9.82, *p* < 0.001, Cohen d = 3.41). In the second analysis, the assumption of homogeneity of variances was not met (F = 7.12, *p* = 0.012), and applying the robust version of the *t*-test did not change the results.

**FIGURE 3:**
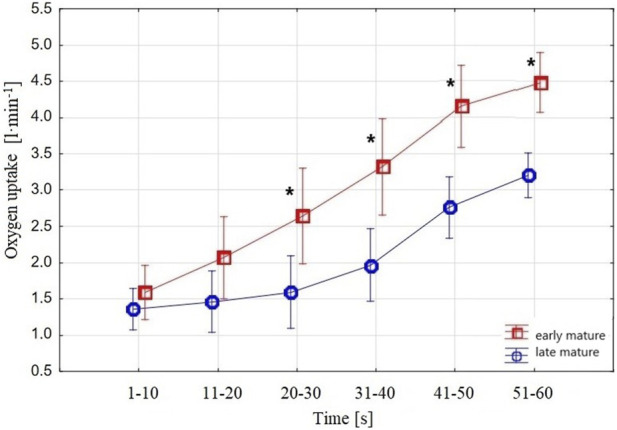
Swimmers’ ${\dot{V}O}_{2}$ (l min^–1^) in the 10-s periods measured in the 1-min tethered test. The upper curve shows ${\dot{V}O}_{2}$ in the *early-mature* group, and the lower curve depicts ${\dot{V}O}_{2}$ in the *late-mature* group. Vertical bars indicate a 0.95 confidence interval; * shows a significant difference between *early-mature* and *late-mature* swimmers.

The older—*early-mature* group had higher ${\dot{V}O}_{2}$ values than the *late-mature* group (Figure 3) (F = 6.75, *p* = 0.02, eta^2^ = 0.17). The two-factor (6 × 2) ANOVA results revealed that ${\dot{V}O}_{2}$ in the 10-s periods was significantly higher in the *early-mature* group of swimmers from the third ( ${\dot{V}O}_{2}$ _21-30_) to the sixth $\left({\dot{V}O}_{2}\right.$ _51-60_) period. The differences (0.23 and 0.61 L min^–1^) between the groups were not significant for ${\dot{V}O}_{2}$ _1-10_ (F = 1.02, *p* = 0.32, eta^2^ = 0.03) and ${\dot{V}O}_{2}$ _11-20_ (F = 3.07, *p* = 0.89, eta^2^ = 0.08), i.e., the first and second periods of the 1-min tethered test.

When analyzing the groups separately, in the *late-mature* group, the ANOVA results showed no difference from the first to the third ${\dot{V}O}_{2}$ 10-s periods (1 vs. 2: F = 0.97, *p* = 0.33, eta^2^ = 0.03; 1 vs. 3: F = 2.01, *p* = 0.16, eta^2^ = 0.06; 2 vs. 3: F = 2.42, *p* = 0.13, eta^2^ = 0.07), whereas in the *early-mature* group, all the 10-s periods were statistically different.

**FIGURE 4:**
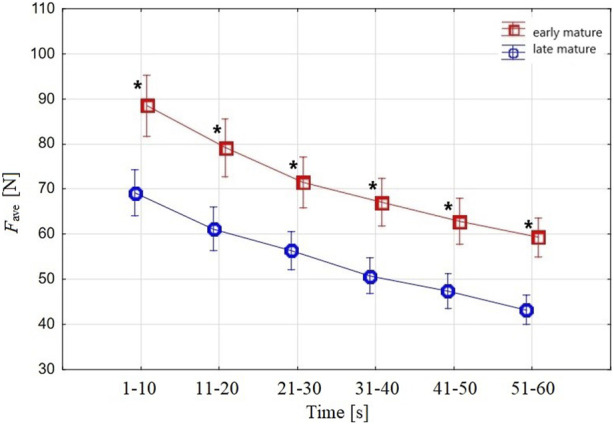
Swimmers’ *F*_ave_ in the 10-s periods measured in the 1-min tethered test. The upper curve shows *F*_ave_ in the *early-mature* group, and the lower curve depicts *F*_ave_ in the *late-mature* group. Vertical bars indicate a 0.95 confidence interval; * shows a significant difference between *early-mature* and *late-mature* swimmers.

*F*_ave_ (Figure 4) in the 10-s periods of the 1-min tethered test was different (two-factor ANOVA, 6 × 2) between the *BA* groups and across the subsequent 10-s periods within the *late-mature* and the *early-mature* swimmers.

**FIGURE 5:**
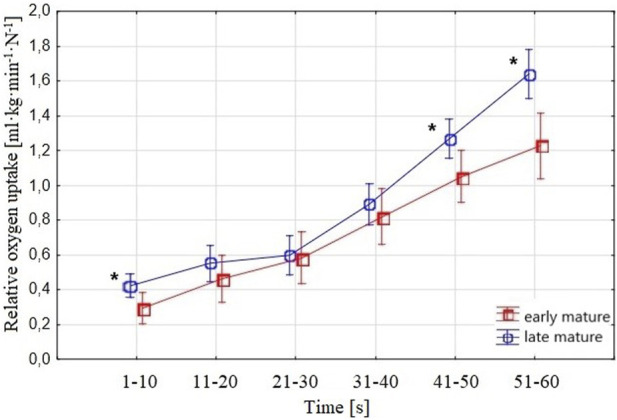
Swimmers’ relative ${\dot{V}O}_{2}$ (ml∙min^–1^∙kg^–1^∙N^−1^) in the 10-s periods measured in the 1-min tethered test. The upper curve shows ${\dot{V}O}_{2}$ in the *early-mature* group, and the lower curve depicts ${\dot{V}O}_{2}$ in the *late-mature* group. Vertical bars indicate a 0.95 confidence interval; * shows a significant difference between *early-mature* and *late-mature* swimmers.

Considering ${\dot{V}O}_{2}$ during the 1-min tethered test, expressed in ml∙min^–1^∙kg^–1^∙N^−1^, (Figure 5), the variance analysis showed a general difference between the *late-mature* and *early-mature* groups (F = 4.82, *p* = 0.011, Cohen d = 0.12). Furthermore, the ANOVA results of the particular 10-s periods (two-factor ANOVA, 6 × 2) between the *late-mature* and *early-mature* swimmers presented no significant differences in the average ${\dot{V}O}_{2}$ values expressed in ml∙min^–1^∙kg^–1^∙N^−1^ for period 2: 0.09 (ANOVA F = 1.10, *p* = 0.30, Cohen d = 0.03), period 3: 0.01 (ANOVA F = 0.02, *p* = 0.90, Cohen d = 0.01), and period 4: 0.07 (ANOVA F = 0.51, *p* = 0.48, Cohen d = 0.01). The differences in average ${\dot{V}O}_{2}$ values in both the *late-mature* and the *early-mature* groups were significant from the first to the sixth 10-s period.

An increased, ranging from low to very strong, correlation was noted between ${\dot{V}O}_{2}$ and *F*_ave_ in the subsequent 10-s periods in the entire group of swimmers (n = 36) (Table 1).

**TABLE 1:** Pearson’s correlations between ${\dot{V}O}_{2}$ (l min^–1^) and *F*_ave_ (N) in the subsequent 10-s periods of the 1-min tethered test in the entire group of swimmers (*n* = 36).

[l∙min^–1^]	${\dot{V}O}_{2}$ _1-10_	${\dot{V}O}_{2}$ _11-20_	${\dot{V}O}_{2}$ _21-30_	${\dot{V}O}_{2}$ _31-40_	${\dot{V}O}_{2}$ _41-50_	${\dot{V}O}_{2}$ _51-60_
	1.44 ± 0.66	1.68 ± 1.03	1.97 ± 1.26	2.45 ± 1.34	3.26 ± 1.19	3.66 ± 0.95
Correlations	0.29	0.40*	0.54**	0.62**	0.79**	0.78**
[N]	*F*_ave1-10_	*F*_*ave*11-20_	*F*_*ave*__21-30_	*F*_*ave*__31-40_	*F*_*ave*__41-50_	*F*_*ave*__51-60_
	76.2 ± 15.08	67.7 ± 14.25	61.9 ± 12.21	56.7 ± 12.11	53.0 ± 11.59	49.0 ± 10.88

* *p* ≤ 0.05; **p ≤ 0.001.

${\dot{V}O}_{2}$ _1-10_ was not significantly correlated with *BA*, the force indices of the 1-min tethered test, and the 400-m front crawl speed or kinematics (Table 2). The subsequent 10-s ${\dot{V}O}_{2}$ periods had a higher relationship with force indices and *SL*. ${\dot{V}O}_{2}$ _21-30_ was highly correlated with the swimming speed (*V*_total_).

**TABLE 2:** Pearson’s correlations between ${\dot{V}O}_{2}$ (l∙min^–1^) in the subsequent 10-s periods and *BA*, entire force indice of the 1-min tethered test, and speed and kinematic indice of the 400-m front crawl race (*n* = 36).

${\dot{V}O}_{2}$	*BA* [years]	*F*_ave1-60_ [N]	*V*_surf_ [m·s^–1^]	*V*_total_ [m·s^–1^]	*SL* [m]	*SR* [cycle∙min^–1^]
	14.0 ± 1.65	60.9 ± 12.19	1.21 ± 0.08	1.29 ± 0.09	1.94 ± 0.23	37.8 ± 4.42
1–10	0.32	0.31	0.17	0.23	0.32	−0.16
11–20	0.50*	0.46*	0.30	0.36*	0.50*	−0.17
21–30	0.62**	0.59**	0.40*	0.44*	0.62**	−0.11
31–40	0.71**	0.66**	0.40*	0.43*	0.71**	−0.07
41–50	0.76**	0.74**	0.36*	0.40*	0.76**	−0.13
51–60	0.81**	0.77**	0.31	0.35*	0.81**	−0.28

${\dot{V}O}_{2}$ , oxygen uptake; *BA*, biological age; *F*_ave_, tethered force; *V*_surf_, surface swimming speed; *V*_total_, 400-m front crawl velocity; *SL*, stroke length; *SR*, stroke rate.

* *p* ≤ 0.05; **p ≤ 0.001.

The subsequent 10-s periods of *F*_ave_ showed a significant moderate relationship with the swimming race results of *V*_surf_, *V*_total_, and *SL* and a strong or very strong relationship with *BA* (Table 3).

**TABLE 3:** Pearson’s correlations of *F*_ave_ (N) in the subsequent 10-s periods with *BA*, entire force indice of the 1-min tethered test, and speed and kinematic indice of the 400-m front crawl race (*n* = 36).

*F*_ave_	*BA*	*V*_surf_	*V*_total_	*SL*	*SR*
1–10	0.77**	0.47*	0.51*	0.60**	−0.32
11–20	0.76**	0.48*	0.51*	0.62**	−0.32
21–30	0.76**	0.52**	0.56**	0.65**	−0.33
31–40	0.80**	0.53**	0.57**	0.59**	−0.26
41–50	0.81**	0.52**	0.55**	0.53**	−0.21
51–60	0.85**	0.51*	0.55**	0.58**	−0.26

*F*_ave_, tethered force; *BA*, biological age; *V*_surf_, surface swimming speed; *V*_total_, 400-m front crawl velocity; *SL*, stroke length; *SR*, stroke rate.

* *p* ≤ 0.05; **p ≤ 0.001.

As depicted in Table 4, there was a significant moderate correlation between the 400-m swimming speed (*V*_surf_ and *V*_total_) and *SL* kinematic and ${\dot{V}O}_{2}$ : ${\dot{V}O}_{2}$ _1-60_ (2.51 ± 0.97 L min^–1^), ${\dot{V}O}_{2}$ _31-60_ (3.15 ± 1.11 L min^–1^), and ${\dot{V}O}_{2}$ _41-60_ (3.47 ± 1.05 L min^–1^). The correlations were strongest with the tethered force (*F*_ave1-60_). *SR* kinematics presented a relationship with *V*_surf_ close to significance (*p* = 0.09).

**TABLE 4:** Pearson’s correlations between *V*_total_, *V*_surf_, and *BA* and *SL*, *SR*, *F*_ave1-60_, ${\dot{V}O}_{2}$ _1-60_, ${\dot{V}O}_{2}$ _31-60_, and ${\dot{V}O}_{2}$ _41-60_ (*n* = 36).

	*SL*	*SR*	*F*_ave1-60_	${\dot{V}O}_{2}$ _1-60_	${\dot{V}O}_{2}$ _31-60_	${\dot{V}O}_{2}$ _41-60_
*BA*	0.54**	−0.43*	0.83**	0.72**	0.78**	0.80**
*V*_surf_	0.33*	*0.29*	0.51**	0.38*	0.36*	0.34*
*V*_total_	0.40*	0.20	0.55**	0.43*	0.40*	0.38*

*BA*, biological age; *V*_surf_, surface swimming speed; *V*_total_, 400-m front crawl velocity; *SL*, stroke length; *SR*, stroke rate; *F*_ave_, pulling force; ${\dot{V}O}_{2}$ , oxygen uptake.

* *p* ≤ 0.05; **p ≤ 0.001.

Additionally, ${\dot{V}O}_{2}$ kinetic variables ${\dot{V}O}_{2}$ _1-60_, ${\dot{V}O}_{2}$ _31-60_, and ${\dot{V}O}_{2}$ _41-60_ were correlated with abilities corresponding with the beginning of a 400-m race, i.e., the first 100-m lap (1.35 ± 0.09 m s^–1^). These coefficients were significant (0.38, *p* = 0.02; 0.36, *p* = 0.03; 0.35, *p* = 0.04, respectively). *BA* showed a strong or very strong relationship with the consecutive variables, but its correlation with *SR* kinematics was negative.

## Discussion

This study aimed to evaluate the oxygen kinetics during a 1-min tethered test in young swimmers with different *BAs*. It was hypothesized that oxygen kinetics might be different in participants with different *BAs*. Calendar age-homogeneous 13-year-old swimmers were significantly differentiated in terms of biological development. Dividing them in accordance with *BA* into *early-mature* and *late-mature* groups revealed their differences in ${\dot{V}O}_{2}$ kinetics (Figure 2). In the 1-min tethered test after the first two 10-s periods of ${\dot{V}O}_{2}$ , the next four 10-s periods were significantly higher in the *early-mature* group than the *late-mature* group. The *early-mature* group revealed a stepwise ${\dot{V}O}_{2}$ increase, with each subsequent 10-s ${\dot{V}O}_{2}$ being significantly higher. The *late-mature* group exhibited a not-significant increase in the first three 10-s periods of ${\dot{V}O}_{2}$ . In the examined 1-min tethered test, the production of a specific swimming pulling force (*F*_ave_) decreased significantly in each 10-s period in both *BA* groups and was higher in the *early-mature* group. The decrease in *F*_ave_ in both groups was almost parallel, although it could be expected in the *late-mature* group; the lower general level of ${\dot{V}O}_{2}$ kinetics, especially in the third period, would enhance this decrease in *F*_ave_. These results suggest, however, that *late-mature* swimmers, owing to their well-trained aerobic power and higher gain in ${\dot{V}O}_{2}$ , were able to bring a slight decrease in *F*_ave_, similar to the *early-mature* peers, presenting a higher $O_{2}$ relative usage (ml∙min^–1^∙kg^–1^∙N^−1^). It should be added that the lack of correlation between ${\dot{V}O}_{2}$ and force, swimming speed, or kinematics and the lack of difference between both groups found in the first 10-s are most probably due to the high initial use of the anaerobic pathway in accordance with the almost instantaneous cardiac output increase, which is initiated by vagal withdrawal and the mechanical pumping action of the contracting muscles in the absence of altered arterial or venous $O_{2}$ contents compared to the level before beginning the test (Poole et al., 2012).

In this study, ${\dot{V}O}_{2}$ in short term,1-min extreme intensity swimming between *late-mature* and *early-mature* swimmers of the same calendar age was compared, which showed that *late-mature* swimmers could be less aerobically economical, requiring a greater percentage of their peak ${\dot{V}O}_{2}$ (per unit of body mass per unit of force). Being aerobically less economical, they were less efficient in the production of pulling force (*F*_ave_), having a less developed or less mature metabolism to produce maximal anaerobic power (Van Praagh and Doré, 2002). The *late-mature* swimmers cannot be as efficient as their older counterparts because of their smaller “metabolic reserve,” as suggested by Bar-Or (1982). This remains in accordance with this study because of similar or higher relative $O_{2}$ usage (ml∙min^–1^∙kg^–1^∙N^−1^) in an extreme swimming effort. The statement in this paragraph may be taken with caution, as early maturing swimmers were heavier and developed much greater thrust.

It can be hypothesized that, generally, a slower increase in ${\dot{V}O}_{2}$ among *late-mature* swimmers during the short-term intense effort (i.e., in the first 30 s of 60 s) was most likely due to a lower percentage of employing or utilizing type II oxidative muscle motor units. This could be ascribed to their recruitment immaturity and to a higher type I muscle fiber contribution to prepubertal to postpubertal boys (Dotan et al., 2012). When the exercise intensity increases, human muscles exhibit organized recruitment adequately to force requirements, which demonstrates that higher-order fibers (i.e., type II) contribute more to force production at higher contraction frequencies (Beelen and Sargeant, 1993).

As explained by Poole et al. (2012), the available evidence supports a greater bias toward oxidative vs. glycolytic metabolism in children compared with adults (in the present study, *late-mature* vs. *early-mature* swimmers). This helps explain a greater percentage of $O_{2}$ usage vs. anaerobic sources in children despite their general lower potential for muscle $O_{2}$ delivery (resulting from a lower cardiac output) at a given work rate and ${\dot{V}O}_{2}$ . Speeding the primary ${\dot{V}O}_{2}$ response is essentially limited to exercise engaging the trained musculature and is generally associated with elevated oxidative enzyme activity within those muscles (Poole et al., 2012). The observed differences in fast oxygen kinetics could be caused by differences in muscle mass (resulting from different *BAs*) and muscle blood perfusion. Greater muscle mass recruitment could potentially impair muscle perfusion, especially during heavy exercise. If muscle perfusion were a limiting factor in ${\dot{V}O}_{2}$ kinetics, this would result in a prolonged fast component when recruiting greater muscle mass (Arend et al., 2021).

In this study, the influence of the ${\dot{V}O}_{2}peak$ on 400-m front crawl performance was examined, and a significant moderate relationship between the ${\dot{V}O}_{2}$ _1-60_, ${\dot{V}O}_{2}$ _31-60_, and ${\dot{V}O}_{2}$ _41-60_ indices and the swimming speed (*V*_surf_ or *V*_total_) was revealed. Studies on competitive swimming have demonstrated that both aerobic and anaerobic metabolisms are important to swimming performance (Zamparo et al., 2000; Figueiredo et al., 2011; Kalva-Filho et al., 2015; Campos et al., 2017). In their meta-analysis of the gathered data, Birat and Ratel (2020) suggested a greater contribution of aerobic energy turnover in 50–400-m swimming races, e.g., 65%–75% and 80%–90% of the total energy turnover is metabolized during 200-m and 400-m races, respectively. In shorter swimming efforts, swimmers require high aerobic power even below 1 min, reaching almost ${\dot{V}O}_{2}\max$ at the finish wall of the race (Costill et al., 1992; Rodríguez et al., 2003; Lätt et al., 2009). On the other hand, Campos et al. (2017) found that 200- and 400-m swimmers’ performance involved higher total values of anaerobic contributions, considering 50-, 100-, and 800-m performance also; both distances (200 and 400 m) can be used to estimate the anaerobic capacity of swimmers and assess changes arising from specific training (Almeida et al., 2021). In addition, Kalva-Filho et al. (2015) demonstrated that total anaerobic capacity was highly associated with 400-m performance and, on the other hand, might be inversely related to the aerobic conditioning level but associated with ${\dot{V}O}_{2}$ slow-component contribution and blood lactate concentration, which should be high (Massini et al., 2023).

It could be stated that short-duration extreme performance is dependent on fast ${\dot{V}O}_{2}$ kinetics (Hellard et al., 2018; Sokołowski et al., 2022a), but this need was also discernible within the moderate exercise intensity domain, requiring 60% and 80% of the gas exchange threshold to produce higher power output in endurance-trained cyclists (Koppo et al., 2004). It was suggested that ${\dot{V}O}_{2}$ kinetics may be attributed to other factors besides $O_{2}$ availability, such as the recruitment of higher threshold motor units. This is in agreement with former studies by Zhang et al. (1991), where ${\dot{V}O}_{2}$ and ${\dot{V}CO}_{2}$ kinetics were faster at each fraction of the work capacity in fitter subjects.

Pulling force results of the all 36 swimmers showed moderate (*I*_ave1-60_) and high (*F*_ave1-60_) correlations with 400-m front crawl performance and a high relationship with the main contributor of swimming speed, i.e., *SL* kinematics, similar to the case of ${\dot{V}O}_{2}$ . Those 400-m main determinants were highly interrelated with the swimmers’ *BA*. This generally remains in agreement with another study, which presents a significant relationship between tethered swimming strength and middle distance front crawl performance (Morouço et al., 2012; Santos et al., 2016; Sokołowski et al., 2022a) and a simultaneous high relationship with ${\dot{V}O}_{2}$ and *BA* (Sokołowski et al., 2022b) and can be related to body composition (Massini et al., 2021; Sokołowski et al., 2023). In the current study, *SR* kinematics, as a basic variable of swimming speed (correlation with *V*_surf_ close to significance, *p* = 0.09), was negatively correlated with *BA* and also with the most prominent determinants of 400-m performance, i.e., *F*_ave_ and ${\dot{V}O}_{2}$ in the subsequent 10-s periods. This can suggest that less mature, *late-mature* swimmers may compensate for lower *SL*, *F*_ave_, and ${\dot{V}O}_{2}$ with a higher rate of stroking, which could be introduced in individualized training. It should also be emphasized that *late-mature* competitors can eliminate the advantage of *early-mature* competitors not only within the surface front crawl swimming zone analyzed in this study but also through techniques such as well-trained dolphin kicks in underwater swimming (Wadrzyk et al., 2021).

In future studies, skeletal muscle volume relating to performance could be calculated to assess the muscle’s effectiveness in producing force during tethered swimming. Considering similar tests closer to the natural conditions, e.g., in a flume, could be seen as an alternative to the static tests carried out in this work, which was its limitation (Ruiz-Navarro et al., 2020).

In conclusion, the 1-min tethered swimming test showed significant differences in the homogeneous calendar age/heterogeneous *BA* groups of swimmers. These were distinguished by a higher level of ${\dot{V}O}_{2}$ kinetics and pulling force indices in *early-mature* swimmers. Lower aerobic power and a less efficient aerobic system in *late-mature* peers were also noted when considering relative $O_{2}$ usage during extreme intensity tethered swimming, with a higher relative $O_{2}$ expenditure per unit of body mass per unit of force (ml∙min^–1^∙kg^–1^∙N^−1^). These differences, such as higher ${\dot{V}O}_{2}$ kinetics and pulling force, were further translated into 400-m front crawl performance, including *SL* kinematics. Swimming training should support the development of these aptitudes in accordance with the *BA* level of swimmers.

## Data Availability

The raw data supporting the conclusion of this article will be made available by the authors, without undue reservation.
